# Ongoing downplaying of the carcinogenicity of chrysotile asbestos by vested interests

**DOI:** 10.1186/s12995-021-00295-2

**Published:** 2021-02-23

**Authors:** Xaver Baur, Arthur L. Frank

**Affiliations:** 1 Chair Em. of Occupational Medicine, University of Hamburg, Hamburg, Germany; 2 European Society for Environmental and Occupational Medicine, P.O. Box 370514, D-14135 Berlin, Germany; 3 Chair Em. of Environmental & Occupational Health, Drexel University School of Public Health, Philadelphia, PA USA

**Keywords:** Asbestos, Asbestos-related diseases, Mesothelioma, Carcinogenicity, Chrysotile, Compensation, Conflict of interests, Public health policy, Regulations, Vested interests

## Abstract

Industries that mine, manufacture and sell asbestos or asbestos-containing products have a long tradition of promoting the use of asbestos, while placing the burden of economic and health costs on workers and society. This has been successfully done in recent years and decades in spite of the overwhelming evidence that all asbestos types are carcinogenic and cause asbestosis. In doing so, the asbestos industry has undermined the WHO campaign to reach a worldwide ban of asbestos and to eliminate asbestos-related diseases. Even worse, in recent years they succeeded in continuing asbestos mining and consuming in the range of about 1.3 million tons annually. Nowadays, production takes place predominantly in Russia, Kazakhstan and China. Chrysotile is the only asbestos type still sold and represents 95% of asbestos traded over the last century.

The asbestos industry, especially its PR agency, the International Chrysotile Association, ICA, financed by asbestos mining companies in Russia, Kazakhstan and Zimbabwe and asbestos industrialists in India and Mexico, continues to be extremely active by using slogans such as chrysotile can be used safely.

Another approach of the asbestos industry and of some of its insurance agencies is to broadly defeat liability claims of asbestos victims.

In doing so they systematically use inappropriate science produced by their own and/or by industry-affiliated researchers. Some of the latter were also engaged in producing defense material for other industries including the tobacco industry. Frequent examples of distributing such disinformation include questioning or denying established scientific knowledge about adverse health effects of asbestos. False evidence continues to be published in scientific journals and books.

The persisting strong influence of vested asbestos-related interests in workers and public health issues including regulations and compensation necessitate ongoing alertness, corrections and appropriate reactions in scientific as well as public media and policy advisory bodies.

## Background

The ongoing promotion of chrysotile asbestos combined with downplaying its adverse health effects in paying compensation continues to be the driven by commercial interests of the asbestos industry and its insurance systems which apply unsound restrictive compensation practices. This paper refers to publications from these vested interests which appeared in recent years in international journals and a book, respectively. The objective is to document false assumptions and predications from these entities using scientific evidence-based facts.

### Questioning of facts, misinterpreting well-established and generally accepted data, manipulating science and unsound changes of diagnostic definitions

#### Promoting chrysotile to the detriment of workers and society with support by affiliated researchers

For decades, the chrysotile asbestos industry has hired scientists for to create the propaganda that chrysotile is safer than amphibole asbestos types and can be used safely [1, 2]. Some of these scientists were also engaged in producing defense material for other industries including the tobacco industry [3, 4]. Egilman et al. [5], by evaluating the published and unpublished studies funded by the Quebec Asbestos Mining Association, including those from researchers at McGill University, identified that data were manipulated and unsound sampling and analysis techniques were used to back up the contention that chrysotile was “essentially innocuous”. As opposed to the overwhelming scientific evidence, affiliated researchers put forth several myths to suggest that chrysotile was harmless, and contended that the contamination of chrysotile with oils, tremolite, or crocidolite was the source of occupational health risk [5]. Even today, several affiliated scientists minimize or even deny the carcinogenicity potency of chrysotile fibers, especially its potency to cause mesothelioma, for example in mechanics servicing car brakes [6, 7]. Some of these publications were used even by the Canadian government to promote the marketing and sale of asbestos, and have had a substantial effect in inhibiting occupational health protection and compensation. The Asbestos International Association (AIA) and today, its successor, the International Chrysotile Association (ICA), now dominated by the Russian chrysotile industry, is promoting chrysotile especially in developing countries [1, 2].

#### Downplaying chrysotile health hazards even in national studies and in scientific publications

Similarly to asbestos industry-financed work by some researchers [8, 9] with frequently undisclosed ties [10–12] also state-sponsored and/or national studies, e.g. from the UK, postulate that chrysotile does not or only rarely cause mesothelioma and other disorders [13] implying that it can be used safely [14]. This is of special concern in developing countries where safety equipment, training, and oversight are mostly lacking. Gilham and colleagues exclusively rely on asbestos fiber counts measured in lung tissue and use this data as an indicator of asbestos dose decades after exposures although chrysotile has almost disappeared due to its low biopersistence. They did not, however, measure fibers in the pleura, where many fibers translocate. These researchers conclude that there is a linear dose–response relationship between the identified remaining lung asbestos burden (of which 98% consisted of amphiboles) and the development of mesothelioma, and that the lung burden should be considered a reliable tool to predict future mesothelioma rates in participants born since 1965. However, only 2% of the fibers identified in the lung were chrysotile, while chrysotile represented as much as 90% of the asbestos used in the UK. As opposed to this interpretation there is well established evidence that chrysotile is able to initiate carcinogenic effects before it disappears from the lung [15–23]. It is also worth mentioning that the carcinogenic potency is not related to the remaining asbestos load in the lung after an interim and latency periods of decades [16, 19, 24–27].

Correspondingly, by use of lung fiber analysis and mesothelioma mortality data from the UK, questioning the carcinogenicity of chrysotile was already done by McCormack et al. [28]. These authors estimated the asbestos-related lung cancer risk by relying on incomplete and/or outdated data and also ignoring the translocation and low lung biopersistence of chrysotile; they made the unfounded and disproved conclusion that mesotheliomas in chrysotile-exposed cohorts are due to other asbestos types. For the severe shortcomings and data misinterpretation in this publication see comment published by Lemen et al. [29].

Our major concern refers to an article issued in a scientific pathology journal [30] and very similarly in an etiology section of a well-established and acknowledged book published by the IARC/WHO, i.e. in chapter 2 [31] where chrysotile carcinogenicity is questioned by selective references such as by quotes from Hodgson & Darnton [32] and Berman & Crump [33, 34] which exhibit significant misclassification of exposure. See especially page 156 in the WHO/IARC book [31] with highly selected citations: *“Some argue that the mesothelial carcinogenicity of chrysotile depends on the level of amphibole fibre tremolite, and that pure chrysotile may not be mesotheliogenic in humans.”* This has been refuted [35].

Specifically, in this etiology section of chapter 2 of the WHO/IARC (page 156), Attanoos et al. reference publications by Berman & Crump [34] and Hodgson & Darnton [32] for the premise that “*Mathematical modeling suggest that mesothelioma incidents is a linear function of amphibole asbestos dose, and a power function of time since first exposure (Hodgson and Darnton (2000), Berman and Crump,(2008)*, *Peto* et al. *(1982* [32, 34, 36]*. There are marked differences in the potency of different fiber types in inducing mesothelioma: commercial amphibole asbestos (amosite and crocidolite) is 2–3 orders of magnitude more carcinogenic than chrysotile (Hodgson and Darnton 2000, Berman and Crump 2003* [32, 33] *… ..) … ..However in sufficiently high doses, chrysotile appears to cause lung cancer and very high doses causes mesothelioma in experimental animals”.* However, animal studies by Wagner et al. [37] and others [38] showed more or comparable figures of cancers from Canadian and other chrysotile sources then from crocidolite.

A related example is “*Exposure response coefficients have been estimated for asbestos from approximately 20 epidemiology studies for which adequate exposure-response data exist. Such coefficients vary widely, however, and the observed variation has not been reconciled. for asbestos exposure*” *among the cited twenty cohort studies Berman & Crump homogenized”* (Berman & Crump, Final Draft: Technical Support Document for Protocol to assess asbestos-related risk, at page 1.2) [33].

Correspondingly, Roggli and colleagues from the Duke University Hospital [39], also engaged in Roggli’s Private Diagnostic Clinic [PDC, Durham NC 27710; which is not mentioned in the publication], although noting that nearly all of their 325 studied mesothelioma cases with informative data had some asbestos exposure [70% household contact, 12% industrial/occupational exposure, 3% building, and 1% environmental exposure] stated that in those 43% where they diagnosed no “objective markers of asbestos exposure” in lungs, that “many of these cases are idiopathic”. Roggli et al. again ignored that even low and short time exposure to asbestos causes mesothelioma [40–44], that there is clearly no threshold in causing this disease, and that chrysotile has a low biopersistence. These authors did not measure asbestos fibers in plural tissue and were not able to detect fibers shorter than five micrometres, although there is strong evidence that the latter are also harmful and are carcinogenic [45–47].

The mentioned assumptions of the authors of the aforementioned WHO/IARC book section and the other publication and of Berman and Crumb are not only based on use of selective and outdated data, in addition, inaccurate, unreliable information about the differences in potency of the different types of asbestos fibers are presented. As given in detail in the next paragraph, mesothelioma risk is not only related to initial, rather also to subsequent asbestos exposures; furthermore, several important studies rebut the assumption of major differences between the various asbestos types.

#### Declining compensation of asbestos victims

Another aspect of disinformation is to broadly defeat liability claims of asbestos victims. A prominent approach for this is systematically denying mesothelioma causation in chrysotile miners and car mechanics repairing brakes and chrysotile miners by Roggli et al. [6, 48–53]. Similarly, insurance affiliated pathologists of the so-called Mesotheliom-Register in Germany have routinely quantified asbestos bodies or asbestos fibers in lung tissue falsely assuming that chrysotile is biopersistent [54, 55]; low chrysotile amounts or its absence in the lung has been broadly interpreted as evidence for a non-asbestos disorder such as idiopathic lung fibrosis in thousands of cases. There is severe criticism on the latter obviously erroneous findings and false interpretations [56–61]. This Mesotheliom-Register, owned and paid for, respectively, by the German statutory accident insurance institutions, has annually given about one thousand expert opinions on the basis of their monopolistic lung pathohistology and fiber examinations on behalf of the statutory accident insurance institutions; they have used for interpreting their findings the unsound restrictive histological and fiber-count-based asbestosis definition by Roggli et al. [62] which Roggli succeeded in introducing into the Helsinki Criteria [63]. For details of this modification of the CAP/NIOSH asbestosis definition and Roggli’s severe conflicts of interest due to his well-paid support by the asbestos industry see [11, 64–69]. Roggli changed early asbestos-induced lesions of grade 1 asbestosis as defined by CAP/NIOSH, to grade 0. Furthermore, and most importantly, without any scientific evidence, Roggli calls for the presence of a certain number of asbestos fibres and/or asbestos bodies in lung tissue as a pathohistological precondition for asbestosis and asbestos-related lung cancer [56, 68]. This latter requirement is especially inappropriate for chrysotile, which comprised about 95% of asbestos used in most Western countries, because of its short half-life in the lung and its limited ability to form asbestos bodies. Subsequently, in Germany the number of accepted lung cancer cases has plateaued at approximately 800 per year for nearly two decades despite the strong linear increase in physician-reported cases amounting to some 5000 annually.

Recently published work by Feder et al. [55] is based on asbestos fiber burden in human lungs and on Roggli’s diagnostic modifications; it fits very well with the studies casting doubt on the well-documented adverse health effects of chrysotile. Their non-substantiated claim that chrysotile fibers are persistent in human lung is contradicted by the collective experience of many experienced research groups [17, 18, 20–23, 70] including the Institute for Occupational and Social Medicine at the Justus Liebig University of Giessen, Germany. In the latter institute lung specimens of more than 350 patients with and 150 without occupational asbestos exposure were analyzed by scanning and transmission electron microscopy and results were interpreted in combination with a detailed clinical and occupational history [71, 72]. Most striking were very low or even missing fibers after a latency period of more than 25 years. In contrast to this well-funded experience noted earlier, Feder et al. reported SEM or TEM fiber analysis based on only six patients at autopsies. Feder et al.’s publication shows several severe shortcomings. It is not clear from which analytical method fiber data was employed and then presented in the supplementary material. The same is true for asbestosis grading, since the authors mention that they followed different heterogeneous definitions. It is important to note that restrictive, unsubstantiated, modified asbestosis grading according to Roggli et al. [62] in combination with unsound high asbestos body or asbestos fiber threshold concentrations has frequently led to denying well-substantiated compensation claims of asbestos victims [65]. Rather than tissue burden, the Helsinki criteria states a history of exposure should be used.

Another approach of German insurance affiliated pathologists was acceptance of low biopersistence of chrysotile, but falsely interpreting its low concentrations or absence in lung tissue in previously exposed workers as having no relevant pathophysiological effects of disappeared fibers by ignoring likely initial disease-initiating effects [16, 73]. It is well known that retained lung asbestos does not predict either lung tumor or mesothelioma risk [50], as also noted above.

A publication by La Vecchia and Boffetta [74] was of special relevance for asbestos compensation issues mainly due to chrysotile exposure in Italy. Industry driven, this publication stated that mesothelioma was caused by initial asbestos exposure (frequently occurring in companies that no longer exist) whereas subsequent exposures were of minor or no causative relevance. This wrong assumption was refuted in several letters and commentaries [43, 75–77]. Asbestos does not only act as an initiator, but has been called a promotor as well, and therefore all exposures matter.

#### Unsound science and policy influence

An important dimension of science and policy malfeasance has arisen with the infiltration by vested interests of governmental decision-making bodies and regulatory processes for assessing hazards, risks and the need for preventive actions. Examples of such conduct include not only asbestos, but also tobacco, pesticides, climate change and many other issues of commercial interest. All these activities have been part of the industries’ campaign to promote their version of ‘sound science’ and ‘good epidemiology’ [78–80]. In so doing, corporate interests frequently have inhibited or even blocked legislative solutions ensuring that public-policy is not based on true sound science [81–83]. There are well-studied examples of corporate manipulation and malfeasance by the asbestos industry, which have influenced the results of scientific findings, delayed important knowledge about the asbestos-cancer relationship, and thereby influenced law and public policy to serve their own interests rather than the interests of workers and public health. Supposedly scientifically credible consultants were engaged to cast doubt on adverse health effects, diagnostic criteria and compensation issues; nowadays, the asbestos industry continues this influence [5, 58, 60, 79, 84–86] by promoting the unrealistic “safe use” of chrysotile asbestos. Their strategy has successfully prevented its banning in various countries and its listing in the Rotterdam Convention declaration [87]. The asbestos industry has published many “product defense” articles, primarily in industry-orientated toxicology journals, mostly written by scientific consultants and consulting firms, or even by ghostwriters; the practice is very similar to the one previously applied by the tobacco, car, food and other industries. Failure to disclose potential conflicts of interests is common for such papers. An example described by the New York Supreme Court Division is the following: Georgia Pacific (GP; which funded a serious of articles, orchestrated and controlled by lawyers, in an effort to create a product defense together with other companies making and selling asbestos-containing joint compounds), misused these studies in its defense of asbestos-related lawsuits. GP entered into a special employment relationship with its director of the Toxicology and Chemical Management to perform expert consulting services under the auspices of its in-house counsel, who also was significantly involved in the pre-publication review-process [11]. Despite this extensive participation, none of the articles disclosed that GP’s in-house counsel had reviewed the manuscripts before they were submitted for publication. Two articles falsely stated that “GP did not participate in the design of the study, analysis of the data or preparation of the manuscript”. As the court remarked, it is of concern that GP’s in-house counsel would be so intimately involved in supposedly objective scientific studies, especially in light of GP’s disclosure denying such participation. The New York court described the consultants’ work, mainly published by Bernstein D.M., Berman D.W. and colleagues as “seeding of the scientific literature with GP-funded studies”. For more details of this practice including ghostwriting see also [4, 10, 11].

To further the myth regarding the “apparent safety” of asbestos, and related products, large scale corruption and distortion of scientific literature took place [88–90]. As described in detail by Egilman et al. [89, 90] and by Michaels [83], asbestos companies have successfully repressed and/or modified the state of the art to cover up the hazards of asbestos.

Egilman et al. [78, 79] particularly addressed the issue of how the asbestos industry and asbestos insurance entities (Metropolitan Life insurance company; MetLife) have influenced compensation laws in numerous states and concocting an arbitrary “protective” standard to monitor asbestos exposure [91]. As stated by Egilman, “MetLife established itself as a public authority in industrial health in the early part of the 20^th^ century, gaining the trust of the public. They were able to use this trust and authority to avoid financial loss, including the firing of sick workers, and avoid legal liability by organizing a network of experts to testify on their behalf in silica- and asbestos-related damage suits. They further manipulated the results of scientific findings from major research institutions, delaying important knowledge about the asbestos-cancer relationship” [79].

## Discussion

The above-mentioned publications of corporate affiliates put forth unreliable illness estimates and conclusions by omitting well established scientific facts, relying on incomplete and or outdated data, or omitting critiques of data sets relied upon, and drawing false conclusions due to use of data sets that are not adequately controlled for latency and/or exposure.

There is also the Vanderbilt talc issue-contaminated with asbestos - where a company official boasted to workers of having a “US Senator in our back pocket”.

As mentioned, there is absolutely no doubt among independent scientists that all asbestos types are carcinogenic and cause many diseases including mesothelioma. This was also IARC’s conclusion upon evaluation of the available literature [92] as well as of other scientific organisations and societies [11, 93–97]. Furthermore, exposure to chrysotile is also associated with the other typical asbestos-caused disorders - asbestosis and plural fibrosis [3, 93–96, 98, 99]. The difference between diseases RRs (risk ratios) of chrysotile versus amphiboles has been debated and is considered by some to be a legitimate but unsettled issue. Some, but not all studies, provide evidence of differences of the various asbestos fiber types. In this connection recent publications by Lenters, Burdorf et al. [100, 101] are worth mentioning. These authors had a closer look at the quality of asbestos exposure assessment; they came to the conclusion that differences between risks of chrysotile and amphiboles are mainly due to shortcomings in exposure assessment than in fiber types.

By summarizing the literature, Lemen [102], Frank [11] and Egilman [103] demonstrate that chrysotile meets all of Hill’s nine proposed criteria of causation, i.e. of malignant mesothelioma. The same is true for the other asbestos-related cancers and for asbestosis. As for other asbestos types, no threshold could be demonstrated below which adverse health effects do not occur.

As mentioned, the counter argument that amphiboles frequently present in very low amounts in chrysotile products are responsible for the disorders found in exposed workers is not likely because the mostly very low or even absent amphibole contamination does not correlate with diseases. Studies in cohorts only exposed to chrysotile fibers such as from specific mines, textile industries, brake repairing, but not to amphiboles, exhibit the typical asbestos-related diseases, especially mesothelioma [27, 104–109]; see also overviews by Lemen at al [29]. and by Lemen 2004 [102]. Frank et al. [35] analyzed Canadian UICC chrysotile B which is free of tremolite by electron microscopy; they found that chrysotile was the only fibrous asbestos component. Very similar results were reported in miners and millers from Balangero, Italy, [104], China [110] or Zimbabwe [106] exposed to amphibole-free chrysotile. In the former East Germany Republic DDR where much Russian chrysotile was used [said to be amphibole free], and sometimes only chrysotile was applied, hundreds of pleural and peritoneal mesotheliomas were recorded and written about [111]. These findings correspond to chrysotile-induced pathology in animal studies which do not support an explanation based on the so-called “amphibole hypothesis”. The animal experiments showed that pure chrysotile is carcinogenic [37, 38, 112–119]. Also in-vitro toxicological studies provided corresponding findings [103].

The aforementioned well-founded outcomes of human and animal studies on adverse health effects of chrysotile as published by the IARC [92], the Collegium Ramazzini [120], and other authors [27, 35, 53, 99, 101, 102, 115, 121–126] were all not cited in recent publications questioning these facts [8, 30, 31, 127]. It has also been ignored that chrysotile, especially its short fibers, move to the pleura where high concentrations can be found in exposed subjects and animals, often with no presence of amphiboles [21, 117, 128–131].

With regard to causative asbestos exposure, it is important that the level of exposure necessary to induce mesothelioma is well below the level necessary to induce asbestosis or other non-malignant asbestos-associated diseases [95]. So it is not surprising that mesotheliomas have been documented not only in occupational settings but also in para-occupational settings such as those occurring among family members exposed to asbestos fibers introduced into the household through the clothes of the worker, and in the vicinity of asbestos manufacturing plants where fiber concentrations are much lower [40, 132–134].

However, it should be mentioned that none of the efforts to use statistical models to characterize relative cancer potencies for asbestos fiber types and sizes have been able to overcome limitations of the exposure data. Quantification of the risk is not reliable because accurate exposure information is lacking for the epidemiological studies used such as Hodgson & Darnton [32] and Berman & Crump [33, 34]. Hodgson & Darnton [135] explained that they relied on “guesstimate(s)” for a number of missing data points [50]. By only referring to studies of earlier potency estimates reported by Hodgson and Darnton [32].

The significantly revised later estimates lowering the potency differences between chrysotile and amphibole asbestos by these same authors (Hodgson and Darnton [135] have been repeatedly ignored as already pointed out by (Lemen, Frank et al. [29]. Resulting uncertainties have been so great that estimates should not be used to drive occupational and environmental health policy. The EPA rejected and discontinued work on its proposed methods for estimating potency factors. For more details see the EPA Report on the Peer Consultation Workshop to Discuss a Proposed Protocol to Assess Asbestos-Related Risk, at pages 3--14.: “*The risk coefficients* (for mesothelioma) *were largely derived from data sets with inadequate exposure--response information for mesothelioma, and assumptions had to be made to determine critical inputs to the mesothelioma model (e.g., average exposure, duration of exposure)”*.

Correspondingly, according to Silverstein, et al. [136], an EPA Peer Consultation Workshop convened to review the 2003 version of the Berman & Crump approach yielded the following criticisms: “*The 2003 report repeated earlier cautions that grossly imperfect exposure characterization in the epidemiology studies creates substantial uncertainties in the estimation of potency factors, including both random and systematic biases. Among the specific data flaws mentioned were unrepresentative sampling strategies, use of surrogate measures in the absence of actual asbestos measure* [136]*.*

## Conclusions

The ongoing promotion of chrysotile combined with unjustified downplaying of its adverse health effects, especially of its carcinogenicity, is driven by commercial interests and is not supported by scientific evidence, see e.g. websites of ICA and its predecessor the Chrysotile Institute [137, 138] and a related commentary [4]. The same is true for widespread restrictive compensation practices based upon lung fiber counts or inadequate risk models [139–141]. As an example, the aforementioned statement of predominantly non-asbestos causation of mesothelioma in women in a chapter in the WHO/IARC book accompanied by similarly distorted statements made by the same or other sponsored or affiliated authors in various journals, lacks scientific evidence and is not true.

A related example is the “amphibole hypothesis” originating from the Quebec industry-sponsored studies. Correspondingly, McCormack et al. [28] and Gilham et al. [13, 142] published that figures showing mesotheliomas related to chrysotile asbestos exposure may be erroneously overestimated, and that mesothelioma in chrysotile-exposed cohorts is due to other asbestos types. As mentioned, the literature shows the opposite [35, 37, 92, 143].

The relative lack of chrysotile biopersistence in the lung combined with its translocation to the pleura and also to the peritoneum and pericardium where the mesothelioma develops has to be taken into consideration when interpreting fiber data in tissue and pathogenicity [24, 128, 131, 144].

The aforementioned influence of vested asbestos-related interests in workers and public health issues including regulations and compensation necessitate ongoing alertness, corrections and appropriate reactions in scientific as well as public media and policy advisory bodies.

It should be mentioned that in general sophisticated mineralogical analysis of lung tissue is a useful method for determining the lung fiber burden when the occupational history does not allow a reliable exposure assessment. However, it has to be taken into consideration that there is considerable interlaboratory variation and it is essential that each laboratory establishes its own reference values [63]. Thus, comparison of data obtained in different laboratories is difficult and this limits the proper use of mineralogical results in the legal field. Furthermore, as stated in the Helsinki criteria [63] at negative non-effect outcome excludes neither potential past asbestos exposure nor the likelihood of asbestos-related disease to develop, while with positive results the possibility of a serious health consequence is increased [145].

## Data Availability

Not applicable.
